# Community diagnostic centres: bringing diagnostics closer to home

**DOI:** 10.3399/bjgp21X717701

**Published:** 2021-11-26

**Authors:** Samuel WD Merriel, Lennard Lee, Richard Neal

**Affiliations:** University of Exeter, Exeter.; University of Oxford, Oxford.; University of Exeter, Exeter.

## BACKGROUND

In October 2021, NHS England announced the creation of 40 new community diagnostic centres in England. The aims are to create faster, more direct access to diagnostic testing, divert patients from hospital to reduce waiting times and the spread of COVID-19, and tackle the backlog of diagnostic activity created by the pandemic. GPs will be able to refer patients to local centres directly for diagnostic tests and reduce the need for hospital outpatient visits. These centres will purportedly be established closer to people’s homes in community hospitals, health centres, repurposed buildings, and even shopping centres, and are planned to be fully operational by March 2022.^1^ Further funding for community diagnostic centres was announced in the Chancellor’s Autumn Statement, taking the total number of centres to be funded to 100, as well as funding for the purchasing of additional diagnostic equipment such as computerised tomography (CT) and magnetic resonance imaging (MRI) scanners.^2^

The drivers for this new initiative stem in part from the NHS Long Term Plan, published in 2019.^3^ Professor Sir Mike Richards, the first NHS National Cancer Director, was commissioned by NHS England to undertake an independent review of NHS diagnostic services following publication of the Long Term Plan, and a key recommendation from his report was the establishment of community diagnostic hubs away from acute hospitals that could be delivered in a COVID-19-safe manner as much as possible. The Richards report also recommended separating acute and elective diagnostics, redesigning diagnostic pathways to better utilise triage tests (for example, faecal immunochemical testing), and a significant investment in diagnostic infrastructure and workforce.^4^

This intervention from NHS England comes in the context of serious chronic challenges with access to diagnostics. England ranks 34th of 38 OECD countries for CT scanners and 31st for the number of MRI scanners per 1 000 000 population.^5^^–^^6^ The NHS has a current diagnostic workforce shortfall of approximately 2000 radiologists,^7^ with a current vacancy rate for radiographers of 10.5% across the UK.^8^ Consequently, pre-pandemic levels of reported GP access to diagnostics in England within recommended National Institute for Health and Care Excellence time frames were low.^9^ The NHS had to adapt the delivery of health care in response to the emergence of the COVID-19 pandemic, and is now left with a significant backlog of patients awaiting ultrasound, CT, MRI, X-ray, and endoscopic diagnostic testing.^10^

There are some clear potential benefits to the new proposed model of community diagnostic centres. Primary care clinicians should be able to refer patients for the appropriate diagnostic test and receive a result they can action sooner, improving care for their patients. Moving diagnostic centres into the community should make access for patients easier through reduced travel, improved parking options and physical access, and greater choice. Separating acute diagnostic activity in hospital settings from outpatient requests by primary care clinicians should, in theory, streamline access in both settings by removing the need for radiology departments to constantly triage requests and re-arrange appointments. Additionally, a more streamlined model for primary care diagnostics could be used for faster implementation of new diagnostic tests in the future.

Evidence suggests that direct access to diagnostic testing for cancer in primary care may be comparable with specialist referral. Friedemann Smith *et al* published a systematic review of diagnostic test use and clinical outcomes in the *BJGP* in 2018.^11^ This study found no significant difference in the pooled cancer conversion rate (number of cancer cases as a proportion of all patients undergoing direct-access testing) or the judged appropriateness of referrals between those originating from primary care clinicians and specialists. Time from referral to test was lower with primary care direct-access testing, and patient and GP satisfaction was high. The majority of included studies were UK based and of poor quality, limiting the confidence in the findings of the review.^11^ Primary care trials of direct-access MRI for knee problems and hysterosalpingography for infertility also found no evidence of a higher number of inappropriate referrals from GPs compared with specialists and reduced waiting times for patients to access testing.^12^^–^^13^

## HOW WILL THE OPENING OF COMMUNITY DIAGNOSTIC CENTRES IMPACT ON NHS WORKLOAD?

The NHS is under very significant workload pressures as a result of the backlogs of care due to the COVID-19 pandemic and high levels of patient demand. General practice is delivering more appointments than ever before, including caring for patients currently waiting for diagnostic tests, outpatient appointments, and specialist treatments. Improving access to diagnostics may address some of the significant waiting times patients are currently facing, allowing diagnoses to be made and treatment commenced in a timelier manner. Patients attending specialist outpatient appointments would also have key investigations already performed, streamlining secondary care. However, diagnostic workforce shortages in the NHS were already significant before the pandemic and it is unclear how these community diagnostic centres would be staffed without a significant increase in radiologists, radiographers, endoscopists, and sonographers in the very near future.

## WHAT ARE THE IMPLICATIONS FOR GPs?

GPs will first need to be made aware of new diagnostic services in their local communities and their remit, and how to access diagnostics for their patients. GPs will be taking on more responsibility for interpreting and acting on reports from diagnostic testing; this is a change that might not be welcome by all in the current context of heavy workloads and a shortage of GPs. Careful selection of patients in primary care to refer for diagnostics will be important to ensure the right patient gets the right test at the right time.

## WHAT IMPACT WILL COMMUNITY DIAGNOSTIC CENTRES HAVE ON LOCAL DIAGNOSTIC PATHWAYS?

Implementation of new community diagnostic centres present an opportunity for innovation in diagnostic pathways. Patient self-referral for testing has been piloted in some areas, such as for chest X-ray in smokers,^14^ and could potentially be considered as part of new models of care. Greater access to diagnostic testing for GPs could speed up diagnoses and reduce the need for some outpatient appointments for patients, or even extend the diagnostic interval for some patients if they ultimately need a specialist referral anyway. Evaluation plans for the new centres are unclear, and are clearly needed to ensure they lead to improvements for patients, clinicians, and the health service.

## CONCLUSION

The creation of community diagnostic centres holds some potential benefits for patients, GPs, and local NHS services. However, successful implementation or value for the NHS is not guaranteed given the huge backlog of diagnostic activity, as a result of the COVID-19 pandemic and longstanding diagnostic workforce shortages, both of which are unlikely to be solved in the short term. The timeline for delivery of these new centres also seems very ambitious given the current challenges faced by the NHS, and may not prove to be deliverable.

## References

[1] (2021). Department of Health and Social Care, NHS England, and The Rt Hon Sajid Javid MP. 40 community diagnostic centres launching across England. GOV.UK.

[2] Treasury HM (2021). Budget and Spending Review — October 2021: what you need to know. https://www.gov.uk/government/news/budget-and-spending-review-october-2021-what-you-need-to-know.

[3] NHS (2019). The NHS long term plan. https://www.longtermplan.nhs.uk/publication/nhs-long-term-plan/.

[4] Richards M (2020). Diagnostics: recovery and renewal — report of the Independent Review of Diagnostic Services for NHS England.

[5] OECD Data Health/Health equipment/Computed tomography (CT) scanners. https://data.oecd.org/healtheqt/computed-tomography-ct-scanners.htm.

[6] OECD Data Health/Health equipment/Magnetic resonance imaging (MRI) units. https://data.oecd.org/healtheqt/magnetic-resonance-imaging-mri-units.htm.

[7] Royal College of Radiologists (2021). Clinical radiology UK workforce census 2020 report.

[8] College of Radiographers (2020). Diagnostic radiography workforce UK census 2020 report.

[9] Nicholson BD, Oke JL, Rose PW, Mant D (2016). Variation in direct access to tests to investigate cancer: a survey of English general practitioners. PLoS One.

[10] NHS England and NHS Improvement (2021). NHS diagnostic waiting times and activity data. Monthly diagnostics data 2021–22.

[11] Friedemann Smith C, Tompson AC, Jones N (2018). Direct access cancer testing in primary care: a systematic review of use and clinical outcomes. Br J Gen Pract.

[12] Wilkes S, Murdoch A, Steen N (2009). Open Access Tubal aSsessment for the initial management of infertility in general practice (the OATS trial): a pragmatic cluster randomised controlled trial. Br J Gen Pract.

[13] Andronis L, Atwell C, Brealey S (2008). Effectiveness of GP access to magnetic resonance imaging of the knee: a randomised trial. Br J Gen Pract.

[14] Kennedy MPT, Cheyne L, Darby M (2018). Lung cancer stage-shift following a symptom awareness campaign. Thorax.

